# Drug-induced interstitial lung disease: a pharmacovigilance study of twelve immunomodulatory and antineoplastic agents

**DOI:** 10.1186/s40780-025-00468-9

**Published:** 2025-07-16

**Authors:** Josef Yayan, Kurt Rasche

**Affiliations:** Department of Internal Medicine, Division of Pulmonary, Allergy and Sleep Medicine, Witten/Herdecke University, HELIOS Clinic Wuppertal, Heusnerstr. 40, 42283 Wuppertal, Germany

**Keywords:** Interstitial lung disease, FAERS, Pharmacovigilance, Amiodarone, Leflunomide, TNF-α inhibitors, Drug safety

## Abstract

**Background:**

Drug-induced interstitial lung disease (ILD) is a potentially severe pulmonary complication associated with various immunomodulatory and antineoplastic agents. Despite increasing recognition, comparative disproportionality data across drug classes remain limited.

**Methods:**

We conducted a retrospective pharmacovigilance study using the FDA Adverse Event Reporting System (FAERS, 2004–2024). Twelve agents with known or suspected associations with ILD were selected. For each drug, the total number of adverse events and ILD reports were extracted. The proportional reporting ratio and reporting odds ratios (RORs) with 95% confidence intervals (CIs) were calculated to assess disproportionality.

**Results:**

Methotrexate and rituximab accounted for the highest number of ILD reports. Amiodarone hydrochloride showed the highest proportion of ILD among its adverse events (9.4%) and the strongest disproportionality signal (ROR = 7.11; 95% CI: 6.79–7.45). Elevated RORs were also noted for leflunomide (3.05), tocilizumab (1.94), pembrolizumab (1.89), and methotrexate (1.90). In contrast, TNF-α inhibitors such as adalimumab (ROR = 0.29) and etanercept (ROR = 0.34) were associated with lower disproportionality signals.

**Conclusion:**

Significant variation in ILD signal strength was observed across drug classes. Amiodarone and leflunomide showed disproportionately strong ILD signals, while TNF-α inhibitors demonstrated lower reporting frequencies. These findings underscore the need for ongoing pharmacovigilance when using agents with potential pulmonary toxicity.

## Introduction

Drug-induced interstitial lung disease (ILD) is a serious and potentially life-threatening condition that can be caused by various medications, particularly immunomodulatory and antineoplastic agents. The clinical presentation and radiological patterns of drug-induced ILD are highly variable, complicating early diagnosis and management [1].

Given the wide range of drugs implicated in ILD, pharmacovigilance studies are essential for characterizing pulmonary safety profiles. The U.S. Food and Drug Administration’s Adverse Event Reporting System (FAERS) is a key resource for detecting and evaluating rare but serious adverse drug reactions in real-world settings [2].

Methotrexate, a widely prescribed immunosuppressive agent for autoimmune diseases, has been associated with pulmonary toxicity, including pneumonitis and fibrosis [3, 4]. Similarly, leflunomide, another disease-modifying antirheumatic drug (DMARD), has been associated with ILD in patients with rheumatoid arthritis [5]. Moreover, immune checkpoint inhibitors—particularly PD-1 inhibitors—have been increasingly associated with ILD, especially in patients undergoing cancer immunotherapy [6].

This study aimed to assess the association between selected of immunomodulatory and antineoplastic agents and the occurrence of ILD using real-world data from the FAERS database. By calculating reporting odds ratios (RORs), we sought to identify drugs with disproportionately frequent ILD reports and contribute to pharmacovigilance efforts aimed at improving pulmonary drug safety monitoring.

## Materials and methods

### Data source

This retrospective pharmacovigilance study used data from the publicly accessible FDA Adverse Event Reporting System (FAERS), a spontaneous reporting database maintained by the U.S. Food and Drug Administration. FAERS collects adverse drug event reports submitted by healthcare professionals, manufacturers, and patients. Reports submitted between January 2004 and December 2024 were included in the analysis.

### Selection of drugs

To ensure a transparent and reproducible drug selection process, we applied a four-step sequential process:

1. Literature Screening (*n* = 45): We initially identified 45 candidate drugs based on published case reports, systematic reviews, and original studies associating them with interstitial lung disease (ILD) or pulmonary toxicity. Searches were performed in PubMed, Embase, and Web of Science [2, 3].
2. Regulatory Safety Review (*n* = 32): These 45 agents were screened for safety warnings and documented ILD risks in regulatory product labels from the U.S. Food and Drug Administration (FDA) and European Medicines Agency (EMA). This reduced the list to 32 agents with explicit pulmonary warnings.
3. Signal Detection in FAERS (*n* = 18): We then screened the FAERS database for a disproportionately high reporting rate of ILD-related adverse event reports. A total of 18 drugs showed pharmacovigilance signals for ILD based on preliminary disproportionality analysis.
4. Clinical Relevance Filtering (*n* = 12): Finally, we prioritized 12 agents commonly prescribed in pulmonary, oncologic, or rheumatologic clinical settings, where ILD poses a clinically meaningful safety concern.

**Fig. 1:**
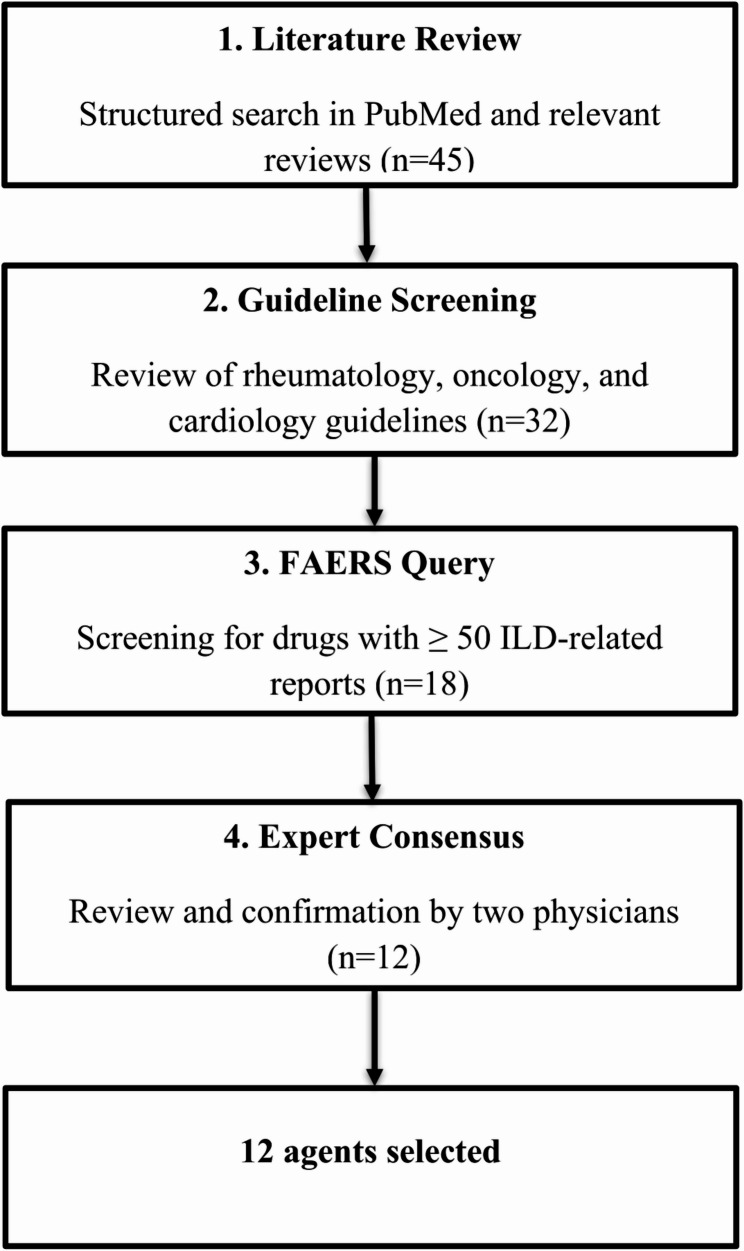
Stepwise selection process of twelve agents for inclusion in the FAERS-based pharmacovigilance analysis. Out of 45 drugs initially identified through literature review, 32 were retained following regulatory safety screening, 18 after confirmation of disproportionality signals in FAERS, and finally 12 were selected based on clinical relevance, as assessed by two physicians

The following 12 drugs were selected for detailed analysis: methotrexate, leflunomide, adalimumab, etanercept, infliximab, prednisone, amiodarone hydrochloride, bleomycin, everolimus, nivolumab, pembrolizumab, and rituximab (Fig. 1). This four-step approach was conducted in a strictly sequential manner, and each subsequent step reduced the pool of candidate drugs. The final set of twelve agents resulted from this narrowing process, as summarized in Fig. 1.

### Definition of ILD cases

 Adverse event reports were included if they contained the term “interstitial lung disease” or equivalent MedDRA terms (e.g., pulmonary fibrosis, organizing pneumonia, pneumonitis). Reports listing one or more of the selected drugs as suspected agents were retained for analysis.

### Data processing and analysis

To evaluate the association between each selected drug and ILD, a disproportionality analysis was conducted. Specifically, we calculated reporting odds ratios (RORs), which compare the odds of ILD reporting for a specific drug with the odds for all other drugs combined. The ROR was computed using a two-by-two contingency table as:

**ROR** = (A × D) / (B × C)​

where A = ILD reports for the drug, B = non-ILD reports for the drug, C = ILD reports for all other drugs, D = non-ILD reports for all other drugs. RORs reflect the disproportionality of reporting and do not represent incidence or causality. This quantifies how much more (or less) frequently ILD is reported for the drug of interest compared to the reporting frequency among all other drugs. RORs indicate reporting disproportionality and should not be interpreted as incidence rates or as evidence of causality. 95% confidence intervals (95% CI) were calculated using the Woolf method. All analyses were performed using VassarStats (www.vassarstats.net) and Microsoft Excel.

## Results

**Fig. 2:**
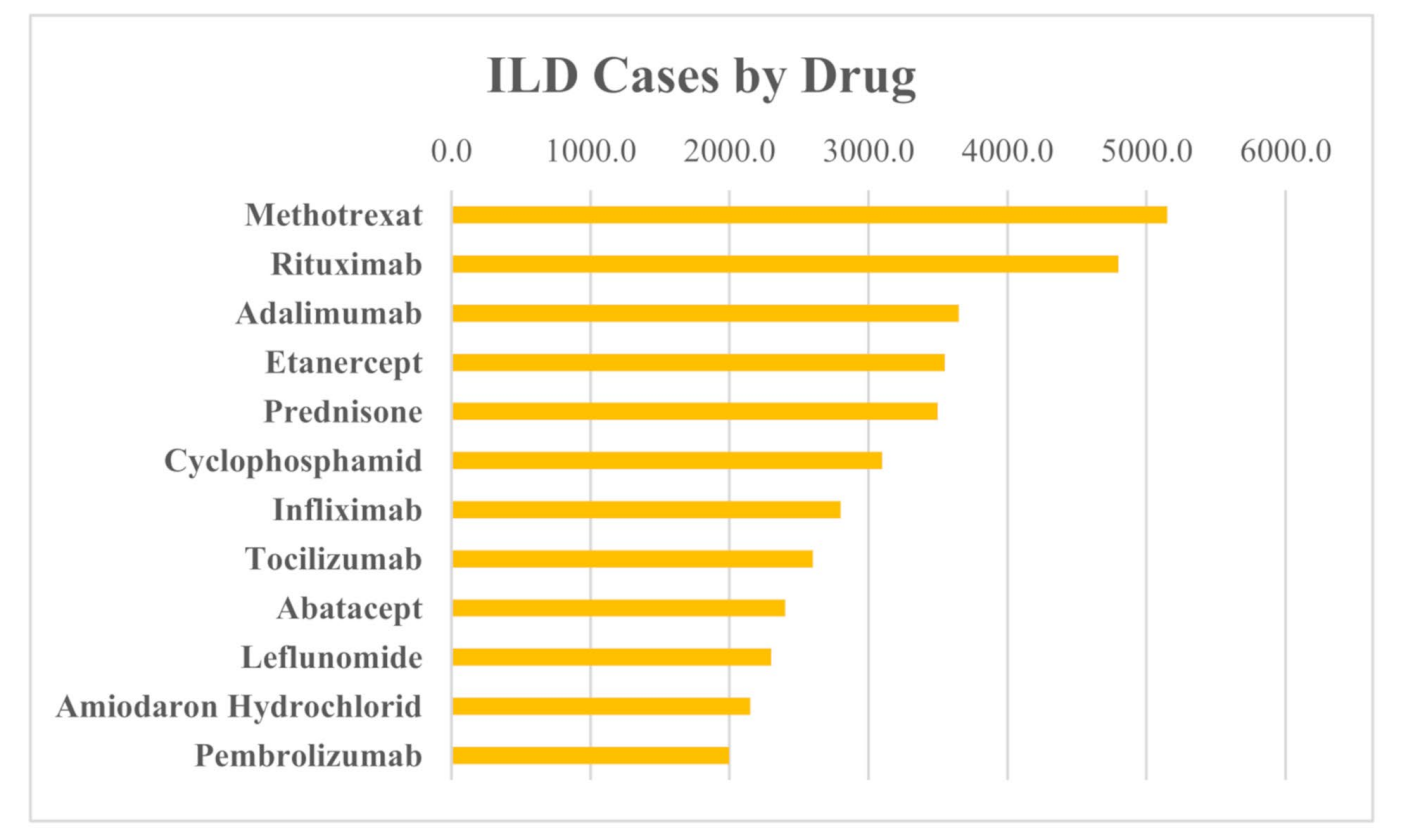
Absolute number of interstitial lung disease (ILD) reports for the twelve selected drugs, based on FAERS data (2004–2024). Bars represent the total number of ILD reports submitted for each drug. Methotrexate and rituximab accounted for the highest number of ILD reports in absolute terms, while amiodarone hydrochloride and leflunomide exhibited the highest proportional ILD reporting frequency relative to total reported adverse events

Twelve drugs commonly associated with pulmonary adverse events were analyzed for interstitial lung disease (ILD)-related reports using data from the FAERS database covering the period from 2004 to 2024. The highest absolute numbers of ILD reports were observed for methotrexate (*n* = 5,132) and rituximab (*n* = 4,778), followed by adalimumab and etanercept (Fig. 2; Table 1). These elevated counts likely reflect the widespread clinical use of these agents rather than a disproportionately signal.

**Table 1:** Summary of adverse event reports and interstitial lung disease (ILD) cases associated with selected drugs, based on FAERS data from 2004 to 2024. For each drug, the total number of adverse events, the number of ILD cases, the ILD proportion (%), and the calculated reporting odds ratio (ROR) with 95% confidence intervals (CIs) are presented. RORs were derived using a two-by-two contingency table, comparing ILD versus non-ILD reports for each drug against all other drugs combined. Amiodarone hydrochloride showed the highest ILD reporting proportion (9.4%) and the strongest disproportionality signal (ROR = 7.11; 95% CI: 6.79–7.45)

Drugs	Total Adverse Events	ILD Cases	Proportion (%)	Reporting Odds Ratio (ROR)	95% CI Lower	95% CI Upper	*P* value
**Methotrexat**	192,935	5132	2.7	1.9	1.84	1.95	**< 0.0001**
**Rituximab**	186,162	4778	2.6	1.81	1.76	1.87	**< 0.0001**
**Adalimumab**	665,965	3688	0.6	0.29	0.28	0.3	**< 0.0001**
**Etanercept**	584,999	3560	0.6	0.34	0.32	0.35	**< 0.0001**
**Prednisone**	167,439	3519	2.1	1.43	1.39	1.49	**< 0.0001**
**Cyclophosphamid**	156,378	3088	2.0	1.34	1.29	1.39	**< 0.0001**
**Infliximab**	200,626	2764	1.4	0.9	0.87	0.94	**< 0.0001**
**Tocilizumab**	92,469	2597	2.8	1.94	1.86	2.02	**< 0.0001**
**Abatacept**	113,095	2434	2.2	1.46	1.4	1.52	**< 0.0001**
**Leflunomide**	55,468	2388	4.3	3.05	2.92	3.18	**< 0.0001**
**Amiodaron Hydrochlorid**	22,410	2116	9.4	7.11	6.79	7.45	**< 0.0001**
**Pembrolizumab**	71,575	1980	2.8	1.89	1.81	1.98	**< 0.0001**

**Fig. 3:**
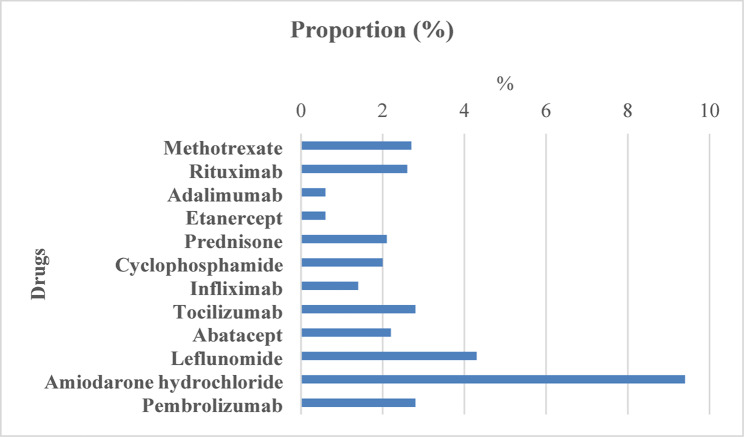
Proportion of interstitial lung disease (ILD) cases among total reported adverse events for each selected drug (FAERS, 2004–2024). Bars indicate the proportion of ILD reports among all adverse events for each drug. Amiodarone hydrochloride (9.4%) and leflunomide (4.3%) exhibited the highest proportional ILD reporting frequencies

To assess the ILD reporting proportion for each drug, the proportion of ILD cases among all reported adverse events was calculated. Amiodarone hydrochloride exhibited the highest ILD reporting proportion (9.4%), followed by leflunomide (4.3%). Intermediate proportions were noted for tocilizumab and pembrolizumab (both 2.8%), methotrexate (2.7%), and rituximab (2.6%) (Fig. 3; Table 1). These proportions suggest a potential pharmacovigilance signal for ILD in the adverse event profile of these drugs.

**Fig. 4:**
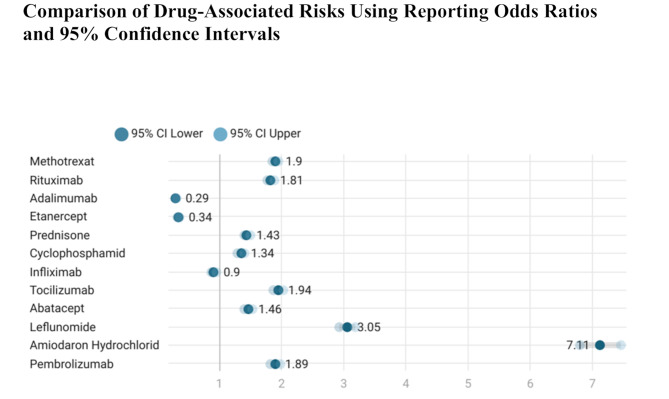
Reporting odds ratios (RORs) and 95% confidence intervals (CIs) for the disproportionality of interstitial lung disease (ILD) reporting associated with each drug, relative to all other drugs (FAERS, 2004–2024). Each dot represents the ROR for a specific drug; horizontal bars indicate the corresponding 95% CI. ROR > 1 indicates increased disproportionality of ILD reporting, ROR < 1 indicates reduced disproportionality. Amiodarone hydrochloride (ROR = 7.11) and leflunomide (ROR = 3.05) exhibited the strongest disproportionality signals; adalimumab (ROR = 0.29) and etanercept (ROR = 0.34) showed the lowest

Reporting odds ratios (RORs) with 95% confidence intervals (CIs) were calculated the disproportionality of ILD reporting for each agent relative to all other drugs in the database. Amiodarone hydrochloride displayed the strongest disproportionality signal (ROR = 7.11; 95% CI: 6.79–7.45), indicating substantially increased odds of ILD being reported in association with this agent. Elevated RORs were also found for leflunomide (3.05), tocilizumab (1.94), methotrexate (1.90), pembrolizumab (1.89), and rituximab (1.81) (Fig. 4; Table 1).

In contrast, adalimumab (ROR = 0.29; 95% CI: 0.28–0.30) and etanercept (ROR = 0.34; 95% CI: 0.32–0.35) were associated with significantly lower RORs, suggesting reduced disproportionality signals for ILD. Infliximab showed a slightly decreased signal (ROR = 0.90), while cyclophosphamide, prednisone, and abatacept demonstrated moderately elevated RORs.

All comparisons yielded highly significant *p*-values (< 0.0001), indicating that the observed RORs are statistically robust and unlikely to have occurred by chance. These results support meaningful patterns of both elevated and diminished ILD disproportionality signals—both positive and negative—regarding ILD reporting across the analyzed agents (Table 1).

Overall, these findings underscore the importance of integrating both absolute case numbers and disproportionality metrics when evaluating drug safety signals. While methotrexate and rituximab contributed the highest number of ILD reports, amiodarone hydrochloride and leflunomide exhibited the strongest individual disproportionality signals, as reflected by their high ILD reporting proportions and markedly elevated RORs. These data may have important implications for clinical decision-making and highlight the value of pharmacovigilance in identifying potential pulmonary safety concerns (Figs. 2, 3 and 4; Table 1).

## Discussion

This retrospective pharmacovigilance study evaluated the disproportionality of interstitial lung disease (ILD) reporting across twelve commonly prescribed immunomodulatory, oncologic, and anti-inflammatory agents using 20 years of post-marketing surveillance data from the FAERS database. The findings reveal substantial variability in both absolute ILD case numbers and reporting odds ratio, as indicated by reporting odds ratios (RORs), across different drugs.

Tocilizumab has demonstrated a comparatively favorable pulmonary safety profile in rheumatoid arthritis patients with ILD. A multicenter retrospective study suggested that tocilizumab may be associated with fewer pulmonary adverse events than other agents in this subgroup [7]. Nevertheless, caution is still warranted, as new-onset ILD or worsening of pre-existing disease has been reported during therapy.

Tumor necrosis factor (TNF) inhibitors have been implicated in both the onset and exacerbation of ILD. A study of 122 cases highlighted the temporal association between initiation of TNF-targeted therapy and subsequent pulmonary deterioration [8]. This is consistent with our findings, where elevated RORs were observed for agents in this class.

The off-label use of biologics in systemic autoimmune diseases has likewise been associated with ILD development. A systematic review by Ramos-Casals et al. documented a broad spectrum of pulmonary complications associated with various biologic agents [9], underscoring the importance of individualized monitoring when initiating such therapies.

Amiodarone, although not an immunomodulatory drug, is a well-established cause of drug-induced ILD. Pulmonary toxicity can occur even at relatively low cumulative doses and is thought to be mediated by phospholipid accumulation in alveolar macrophages [10, 11]. In our analysis, amiodarone showed one of the highest ILD case counts and the strongest disproportionality signal.

Checkpoint inhibitors, including nivolumab and pembrolizumab, have become increasingly associated with immune-related pneumonitis. While early meta-analyses reported incidence rates of up to 4% [6], subsequent real-world studies have confirmed ILD as a relevant clinical concern [12]. Our FAERS-based results align with these observations, showing a high ROR for nivolumab (2.59; 95% CI: 2.43–2.75).

Rituximab, a B-cell–depleting monoclonal antibody, has also been associated with ILD. Although rare, severe cases of rituximab-induced pulmonary toxicity have been described [13]. In our study, rituximab exhibited a moderately elevated ROR of 1.35 (95% CI: 1.20–1.52).

Drug-induced ILD shows histopathological heterogeneity. Prior reviews have shown that interstitial patterns vary by drug, ranging from organizing pneumonia to diffuse alveolar damage [14, 15], further complicating clinical attribution and diagnosis.

Taken together, these findings emphasize the need for individualized pharmacovigilance in high-risk patients—particularly those with pre-existing lung disease or receiving multiple immunomodulatory therapies. Although spontaneous reporting systems such as FAERS are inherently limited by underreporting, missing data, and the absence of exposure denominators, they remain valuable tools for detecting real-world pharmacovigilance signals.

## Limitations

This study has several limitations inherent to spontaneous pharmacovigilance databases such as FAERS. First, underreporting and reporting bias are common, particularly for subclinical or non-serious ILD cases, while overreporting of well-known adverse events may distort signal detection. Second, causality cannot be established due to the lack of detailed clinical context and uncertain temporal relationships between drug exposure and event onset. Third, FAERS does not provide reliable denominator data (e.g., the total number of exposed individuals), precluding the calculation of true incidence rates. Therefore, the reporting odds ratios (RORs) presented here should be interpreted strictly as disproportionality signals—reflecting relative reporting frequencies—not as measures of actual risk. Fourth, misclassification bias may arise from variability in coding practices or ambiguous diagnostic terminology. Despite the use of an inclusive MedDRA term set, relevant ILD cases may have been missed or misclassified. Lastly, heterogeneity in reporting practices across drugs, therapeutic classes, regions, and time periods may introduce confounding effects that limit cross-drug comparability.

## Conclusion

This pharmacovigilance analysis of FAERS data revealed considerable variation in the reporting proportion and disproportionality disproportionality signal of ILD across commonly used immunomodulatory, oncologic, and anti-inflammatory drugs. While methotrexate and rituximab accounted for the largest number of ILD cases in absolute terms, amiodarone hydrochloride and leflunomide exhibited the strongest reporting signals, both in terms of ILD proportion and reporting odds ratios (RORs). Conversely, TNF-α inhibitors such as adalimumab and etanercept demonstrated comparatively low disproportionality signals for ILD. These findings underscore the importance of integrating both absolute case numbers and disproportionality-based indicators when assessing pulmonary safety profiles. Given the limitations of spontaneous reporting systems, our results should not be interpreted as evidence of causation. Nonetheless, these data provide valuable real-world insight into potential ILD signals and support the need for vigilant monitoring, especially when initiating agents known to affect lung function. Future prospective studies and registry-based analyses are warranted to validate these findings and better inform clinical risk–benefit decisions.

## Data Availability

The datasets generated and analyzed during the current study are available from the FDA Adverse Event Reporting System (FAERS): https://www.fda.gov.
